# Collaborative collection development: a MedPrint case report

**DOI:** 10.5195/jmla.2023.1373

**Published:** 2023-07-10

**Authors:** Wyoma vanDuinkerken, Zachary Valdes

**Affiliations:** 1 wvanduin@library.tamu.edu, Director of the Joint Library Facility, Texas A&M University – RELLIS Campus, Bryan, TX 77807.; 2 zach.valdes@shsu.edu, Associate Professor, Head of Cataloging and Metadata, Newton Gresham Library, Sam Houston State University, Huntsville, TX 77341.

**Keywords:** Medical periodicals, shared collection, medical retention program

## Abstract

**Background:**

In response to several of Texas' largest medical libraries being forced to discard all serial print holdings, the Texas A&M University System and University of Texas System's Joint Library Facility (JLF) staff worked to help provide a solution to save and store these resources. This process fire-started a comprehensive effort by JLF staff to contact the National Library of Medicine (NLM) and devise a blueprint that would be used to help save and preserve all serial medical resources listed in NLM's medical retention program.

**Case Presentation:**

In an unprecedented approach, the Texas A&M JLF staff launched efforts to collect and preserve the complete holdings range of all NLM MedPrint periodical runs. This case report details the planning and steps JLF staff took to accomplish this feat; highlights important matters of consideration for the medical community which heavily relies upon continuous access to MedPrint materials; and provides insight on the apparent preservation vulnerabilities these materials increasingly face in an environment where digitization may create a false sense of security.

**Discussion:**

By May 2021, JLF had collected complete title runs up to year 2000 for 202 of the 254 MedPrint titles, which consists of more than twelve thousand volumes. These efforts proved particularly beneficial in the wake of the COVID-19 pandemic, which forced NLM to halt ILL processing from their print collection. During this time, JLF was uniquely positioned to meet and respond to the historic high number of medical literature ILL requests it received during this time.

## BACKGROUND

In 2011, National Library of Medicine (NLM), in conjunction with its Network of the National Library of Medicine (NNLM) developed and implemented a national cooperative collection management program aimed at safeguarding the “preservation of and continued access to the literature through a national cooperative medical serials print retention program” [1]. The idea was that this voluntary program would encourage the members of NNLM, which consists of academic health science libraries, hospital, pharmaceutical and other special biomedical libraries, public libraries, information centers and community-based organizations, to work together to retain approximately 250 serial titles for future use. The identified list of journals to retain was developed in consultation with the Regional Medical Libraries and used two key resources, Abridged Index Medicus and PubMed Central, as its foundation. In September 2011, MedPrint was launched, and libraries began identifying which titles they would retain [2]. As Carrigan et al. note, by August 2016 medical libraries had agreed to retain all but three of the MedPrint journal titles [3].

According to Fishel and Collins, one of the reasons that led to this collaborative collection management plan was that medical “libraries increasingly have come under pressure to give up physical space to other areas of their parent organizations” [4]. As a result, libraries have had to discard an increasing number of print journal titles, limiting patron access to the years and volumes available online. Two regional task forces further confirmed this trend when they reported that libraries were “facing pressure to reduce or repurpose library space have already begun removing back-issue print journal collections from their stacks” [2].

One way that medical libraries addressed this challenge was to transition their print journal subscriptions to electronic format, which helps alleviate space constraints while continuing to provide serial access to patrons. Additionally, publishers began converting their “print only” journal volumes to electronic format, thereby creating a virtual library of backfiles libraries were able to purchase. However, as Fishel and Collins stressed, not all titles in a publisher's portfolio are being scanned, and there is also no guarantee that all the volumes and issues of a selected title designated to be scanned will be converted to electronic format [4]. Further, even when volumes are scanned, these electronic surrogates are frequently susceptible to quality issues that may inhibit patrons' use of the materials, such as “missing content (volume issues or pages), poor-quality images, and illegible text from poor-quality scans.” [5]. Adding to the concern of incomplete scanning was the uncertainty around some publishers' long-term electronic backfile preservation plans. As a result, a growing concern emerged that as libraries began removing print from their collection, they were becoming vulnerable to unanticipated preservation deficiencies [2].

The goal of the MedPrint program was to alleviate these quality and preservation concerns by having at least twelve print copies of all the MedPrint title runs retained in libraries around the United States, with an additional copy being held by NLM. If libraries agreed to this commitment and signed the agreement, they would need to keep these titles until September 30, 2036, a 25-year retention commitment. Once both the library and the NLM signed the agreement, the contract would go into effect until being subject to renewal upon the completion of the term [6].

However, as libraries faced an increasing amount of pressure to give up their traditional stacks space to repurposing efforts, an expectation developed that health sciences libraries would begin moving their print collections to coordinated or shared management facilities [7].

## CASE PRESENTATION

In 2013, the University of Texas System and Texas A&M University System opened the Joint Library Facility (JLF), a storage facility meant to hold a collaboratively owned collection. The JLF has become one of the largest cooperative high-density storage facilities in the state of Texas [8]. Located on the campus of Texas A&M University in College Station, Texas, this high-density storage facility houses materials by size and in trays on oversize shelves which are 36 inches deep by 54 inches wide. The JLF shared collection facility concept provides a unique service in that it operates not just as a storage facility, but as a shared collection in which all participating libraries operate under a Resource in Common (RIC) model [9]. The RIC model ensures that all participating libraries share ownership of the content within the collection and may request access to said content through Interlibrary Loan (ILL) requests. As more materials have been submitted to the facility through a vigorous accession process, the collection has continued to grow at a rapid pace and is now on the verge of finalizing its third and final storage unit, altogether housing more than one million volumes.

Prior to the facility's opening, one of Texas's largest medical libraries was forced to discard all of its print holdings. This was the first of several medical libraries that turned to the JLF for help with saving medical material. For JLF staff, a real concern developed that medical serial titles which were listed in MedPrint, the national cooperative medical serials print retention program, may be lost as Texas medical libraries faced increasing pressure to give up their physical stack space. In hopes of addressing this concern, the director of JLF in 2015 contacted NLM to discuss the prospect of preserving the entire MedPrint collection within the walls of JLF. This case study will discuss issues and the process taken to collect these items, and how far along the project has progressed.

This longitudinal case study uses quantitative data derived from bibliographic information (e.g., participating library catalogs), archival software (ARCHON), and online catalog software (Library Inventory Search Assistant) to track the steady progress of ingestion of MedPrint volumes, and to identify any incomplete or missing volumes. Using these data, JLF staff generated a list of items meeting the aforementioned criteria and began systematically reaching out to participating libraries in an attempt to acquire as much of the designated content as possible to facilitate future preservation.

The first step JLF staff needed to take was to discover if JLF was eligible to participate in the NLM MedPrint program. There was a concern that since it was not a designated medical library or solely a medical library storage facility, JLF would not be able to participate. To verify this point, the JLF director referenced the MedPrint participant agreement, which confirmed eligibility in its list of requirements, which stipulated that U.S. library DOCLINE participants were eligible to serve as MedPrint partners, but that these libraries must agree to hold and retain full runs of selected titles, with those holdings being at least 95% complete [6].

Since its inception, JLF administration invested in being a DOCLINE participant and OCLC sharing partner so that JLF could provide MedPrint titles to ILL requestors, which satisfied the first requirement. Additionally, at JLF's onset, JLF administration's aim was to collect all complete volumes of each title found on the MedPrint list, including all past, present, and future volumes, and including those published beyond 2000. As a result, the second and third NLM requirements did not pose any issues for the facility.

Additionally, there were six multipart requirements that participating MedPrint libraries would need to agree to fulfill once they determined that they could participate in the program. These requirements included items such as retaining the original print format until September 30, 2036; verifying holdings are at least 95% complete at the volume level; and confirming that the general condition of the held volumes are in usable condition. Further, the requirements called for the items to have accurate holdings maintained in DOCLINE with annual accuracy reviews; and that participating institutions would allow these holdings to be exported from DOCLINE to serve other registry and database needs. The remaining requirements pertained to actions that would help ensure long-term preservation and access of the materials, such as requiring that the materials are stored in climate-controlled facilities with disaster prevention functionality and that they would be accessible to onsite patrons and ILL requestors.

The decision to become an NLM MedPrint participant ultimately required review by Texas A&M University Libraries Administration and was approved with the understanding that this process would be completed in separate phases over multiple years. The first phase would involve collecting all needed volumes over one to two years, while the second phase would involve verifying each journal had all their parts in place, including covers, tables of contents, advertisements, and supplements.

JLF staff began by reviewing the official list of 254 MedPrint titles. Although the years of publication were given, libraries needed to agree to retain the title beginning with the first published volume until the title ceased in print. If the title did not cease and was still being published in print, libraries would need to agree to keep the print volumes of those titles for publication dates ranging through at least the year 2000. The JLF staff's goal was to commit to collecting and keeping all the print volumes, even if they extended past the publication year of 2000. Additionally, it was decided that even if participating libraries did not have the needed volumes, JLF staff would actively purchase any volume needed up to publication year 2000, but would not actively purchase any volume past 2000. This decision was made to ensure the program titles were preserved in a full and comprehensive state where possible.

As JLF staff began validating JLF's holdings against the MedPrint list in 2015, they discovered that JLF already had a significant number of volumes for these titles. Of the 254 titles on the list, there were 139 titles that had ceased and were no longer being published, and 98 that were still being published. For the remaining 17 titles, JLF staff discovered that some publication status information conflicted with what was observed in both OCLC and DOCLINE. For example, MedPrint showed some titles as still being produced, while OCLC showed they had ceased. Some publications' beginning dates had discrepancies. The JLF staff elected to err on the side of caution and record bibliographic information at the volume level according to what was found in DOCLINE and OCLC.

**Figure 1:**
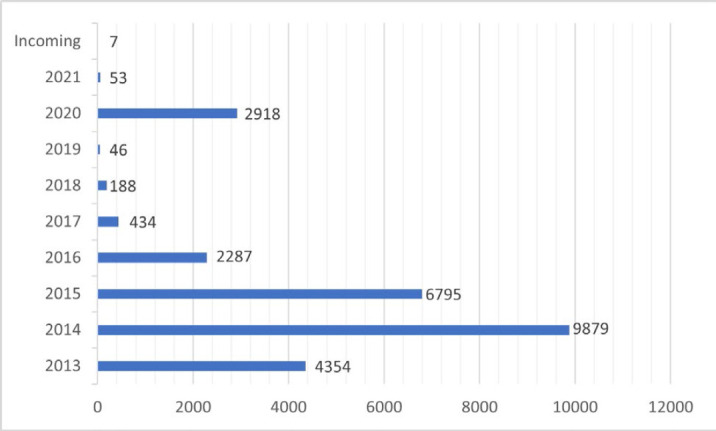
MedPrint Volumes by Year Ingested into JLF

By the end of 2020, JLF held 26,901 volumes of the collection, and had 60 additional volumes ingested or designated to be housed in JLF in 2021 (See Figure 1).

Additionally, JLF staff sought to collect complete title runs whenever possible (i.e., collecting all published volumes for any given periodical title). By May 2021, JLF had collected complete title runs through the publication year 2000 for 202 of the 254 MedPrint titles, which consisted of 12,307 volumes. This left only 423 outstanding volumes to hold complete title runs for all 254 MedPrint titles through the publication year 2000. When broadening the complete title run criteria to all published volumes regardless of year (i.e., even volumes published after the year 2000), JLF collected complete title runs for 170 of the 254 MedPrint titles, which consisted of 13,999 volumes. However, JLF is a low-use facility, meaning that it receives materials with low circulation averages as defined by each individual participating institution, and it does not receive materials currently being published. Therefore, it would be unlikely for the facility to acquire the latest or most current published volumes making up the remaining 84 titles. As a result, a decision was made to stop the volume count at the end of the run that JLF held, and not at the end of the run for ongoing publication titles. This meant that if JLF had a MedPrint title that ran from 1990 to 2021 (ongoing) but JLF only held 1990 to 2019, the facility would see JLF's holding range of 1990 to 2019 as a full run of the title. The remaining volumes needed for 2020 onwards would then be sought at a future time when they were no longer considered current and in high use.

The JLF staff then updated JLF's holdings in DOCLINE to reflect these latest additions for complete accuracy. Although adding these titles and volumes to DOCLINE was required annually, JLF's internal policy was to have the participating libraries' DOCLINE updated after the participating library deposited items into the facility. The JLF staff decided that they would double check the accuracy for each deposited title and ensure that permanent retention was placed on all MedPrint volumes within DOCLINE in January of each year.

Similar to the initial acquisition process, JLF staff developed a system in which they utilized bibliographic information, archival software, and online catalog software to track the steady progress of ingestion of MedPrint volumes, and to identify any incomplete, or missing volumes along the way. In tracking this data, JLF staff were able to generate a live list of this content and began systematically reaching out to participant libraries in an attempt to secure missing content. Due to the preference to have as little impact on existing medical library collections as possible, JLF staff elected to first contact non-medical libraries before contacting medical libraries when attempting to acquire any potential missing volumes. As a last resort option, JLF staff sought out purchase options for any final remaining volumes.

Upon finalizing these procedures, JLF staff began contacting JLF's participating libraries who held the needed MedPrint volumes to first determine whether these items were in low use to their libraries, and whether they were willing to send them to JLF for the MedPrint program. For those rare items that could not be found, JLF staff began purchasing them from available online merchant venues for future generations. To accomplish this, the Acquisition department of Texas A&M University searched seller sites for the titles JLF did not have. During this process, 151 volumes from 16 titles were found and purchased. While JLF staff's general approach at this stage was to not purchase any volume published after the year 2000, 6 volumes with post-2000 publication dates were ultimately purchased when conveniently located during this stage. Once all MedPrint volumes are secured and in possession of the facility, JLF administration will file paperwork with NLM to officially register all volumes in the system.

**Figure 2:**
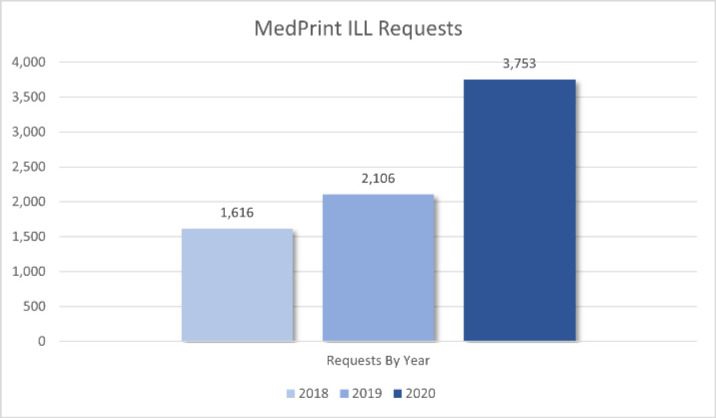
MedPrint Volumes ILL Requests by Year for JLF

Coincidentally, the timing of JLF collocating MedPrint titles proved to be fortuitous in helping to ensure these materials remained accessible to the medical community amidst the unanticipated logistical challenges introduced by the COVID-19 pandemic. For example, in response to difficulties resulting from COVID-19, NLM was forced to halt ILL processing from their print collection from March 2020 to September 2020. During this timeframe, JLF staff saw historic highs in medical literature ILL requests with an increase of 78.2% in 2020 (3,753) over the previous high from 2019 (2,106). Due to these collocation efforts, JLF was strategically positioned to provide this service at a time when few other institutions could (See Figure 2).

## DISCUSSION

Although MedPrint was initially established as an effort to preserve valuable print medical periodical content, few could have anticipated how the increasing demand for space within the library environment would threaten the preservation commitments this program was built upon. Indeed, libraries have long been cognizant of the reality surrounding increased efforts from parent institutions to reduce or repurpose libraries' physical space. However, the occurrence of multiple libraries reneging on MedPrint retention commitments prior to the program's halfway point may indicate that libraries have either underestimated the pace and pervasiveness surrounding these types of reclamation efforts, or perhaps overestimated the robustness collaborative retention efforts, such as MedPrint, pose against these pressures when institutional support is not firmly in place. As libraries grapple with the bleak reality of shrinking library space and its potential impact on print retention, JLF's efforts have presented a viable alternative that ultimately helps position these libraries to ensure continued ownership, access, and long-term preservation of at-risk MedPrint materials. Further, the methods and structure described here may serve as an initial model for other institutions who wish to begin their own efforts towards ensuring long-term retention of high-value print materials which may also be at high risk of being discarded due to similar pressures.

Additionally, this service fulfillment further demonstrated the value of collaboration between academic libraries (both medical and non-medical) and shared print repositories in providing copious medical literature ILL alternatives to offset unanticipated events, such as the NLM closures that occurred due to COVID-19.

In moving forward, JLF staff aims to assess the remaining obstacles that other institutions could find themselves encountering when engaging in similar retention efforts. The first of these obstacles involves addressing acquisition efforts for any remaining unsecured MedPrint materials. While JLF staff have been able to secure the vast majority of content originally assigned to this preservation objective, there remains a small percentage of content that they have not yet been able to secure but continue to pursue. This includes pre-2000 print content, which may ultimately require identifying purchasing opportunities through which these materials may become available to JLF. However, titles included in this program which are still being published may pose new obstacles in the future as the industry progressively moves away from the print format, and as the acquisition of print content consequently becomes more challenging due to lower overall production volume. Even so, JLF staff have elected to focus on continuing JLF's retention efforts for any MedPrint title still in publication by revisiting them every five years to determine the number of print volumes published over this timeframe, and to assess the feasibility of acquiring this content. Additionally, JLF staff will monitor advancements in the electronic preservation of print materials—particularly with regard to scan quality, scan completeness, long term backfile preservation arrangements, and shared rights challenges—in hopes that these advancements may provide a new means for these materials to be more effectively archived in electronic formats for future generations of medical researchers.

## Data Availability

Data associated with this article are available in the Joint Library Facility's repository at https://library.tamu.edu/joint-library-facility/spreadsheets/Medprint%20final%20numbers%20as%20of%207%2027%202021.xlsx.
